# Supplementary figures and images for: Moisture as a key factor alleviating low-temperature stress: Effects of hydrothermal conditions on maize emergence
Source: PLoS One. 2026 Feb 23;21(2):e0340773. doi: 10.1371/journal.pone.0340773 (PMC12928403; doi:10.1371/journal.pone.0340773)

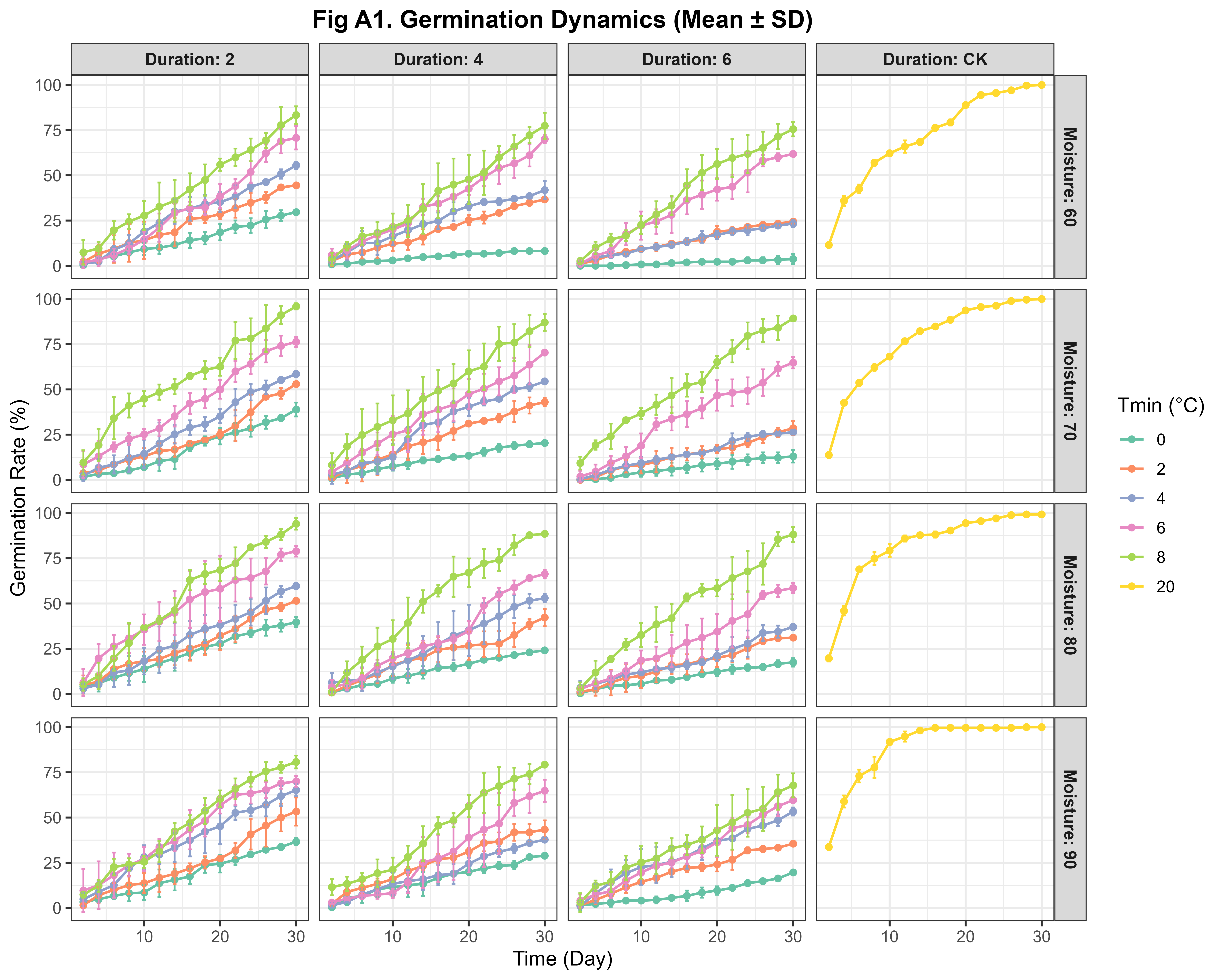

Supplement: S1 Fig A1 — Cumulative germination (%) over time in maize seeds subjected to different minimum temperatures (Tmin: 0 ~ 8°C), chilling durations (2,4,6 h), and soil moisture levels (60%,70%,80%,90% field capacity).Each curve represents the mean of three replicates (n = 90), with error bars indicating standard deviation. The control (CK) group was maintained at 20°C for 24 h without chilling stress. (TIFF) [file pone.0340773.s001.tiff]

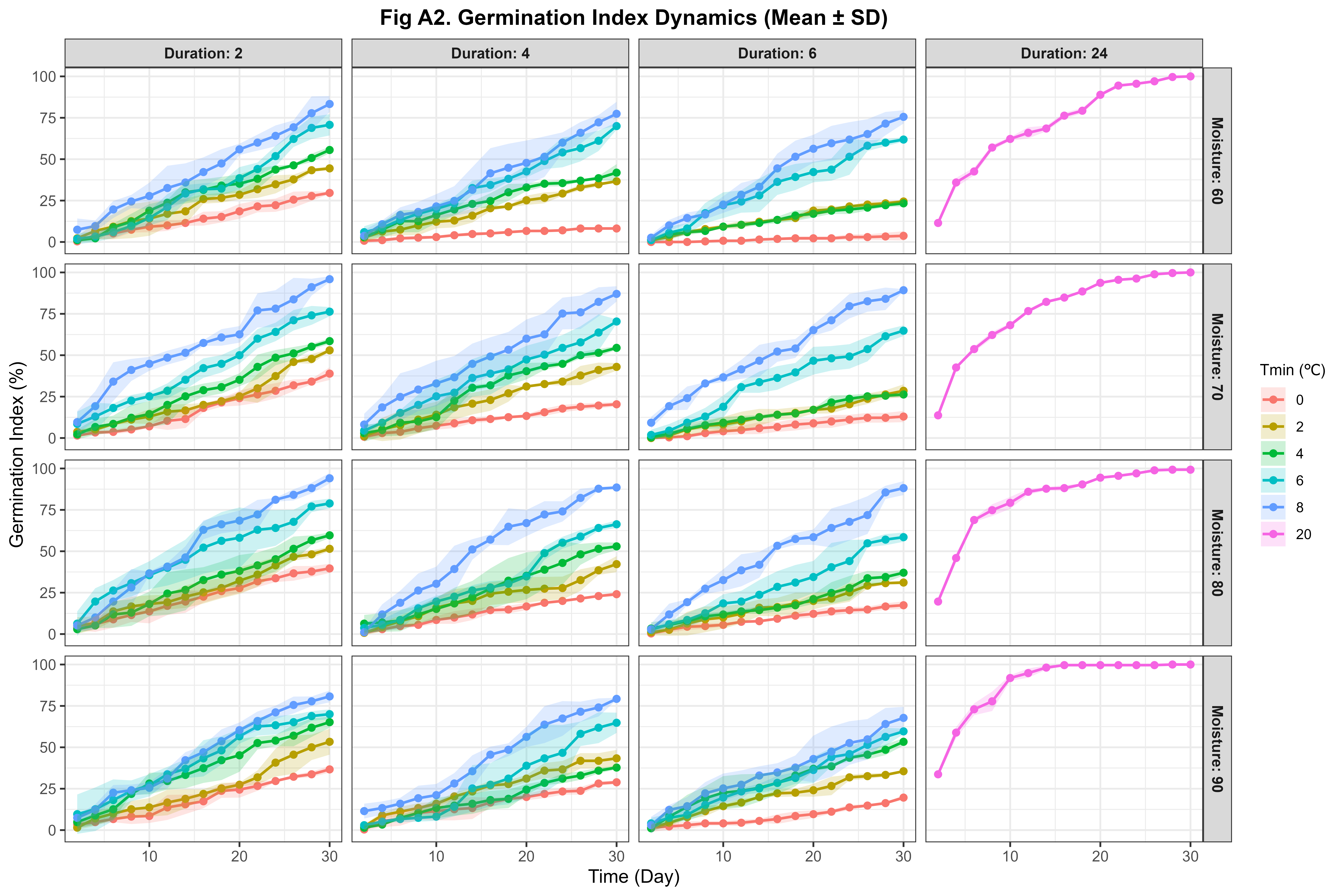

Supplement: S2 Fig A2 — Daily germination index (%) of maize under various chilling temperature, duration, and moisture combinations. Each point reflects the average value from three replicates. Lower Tmin and prolonged duration suppressed the index, while higher soil moisture (80 ~ 90%) accelerated accumulation under moderate cold stress. (TIFF) [file pone.0340773.s002.tiff]

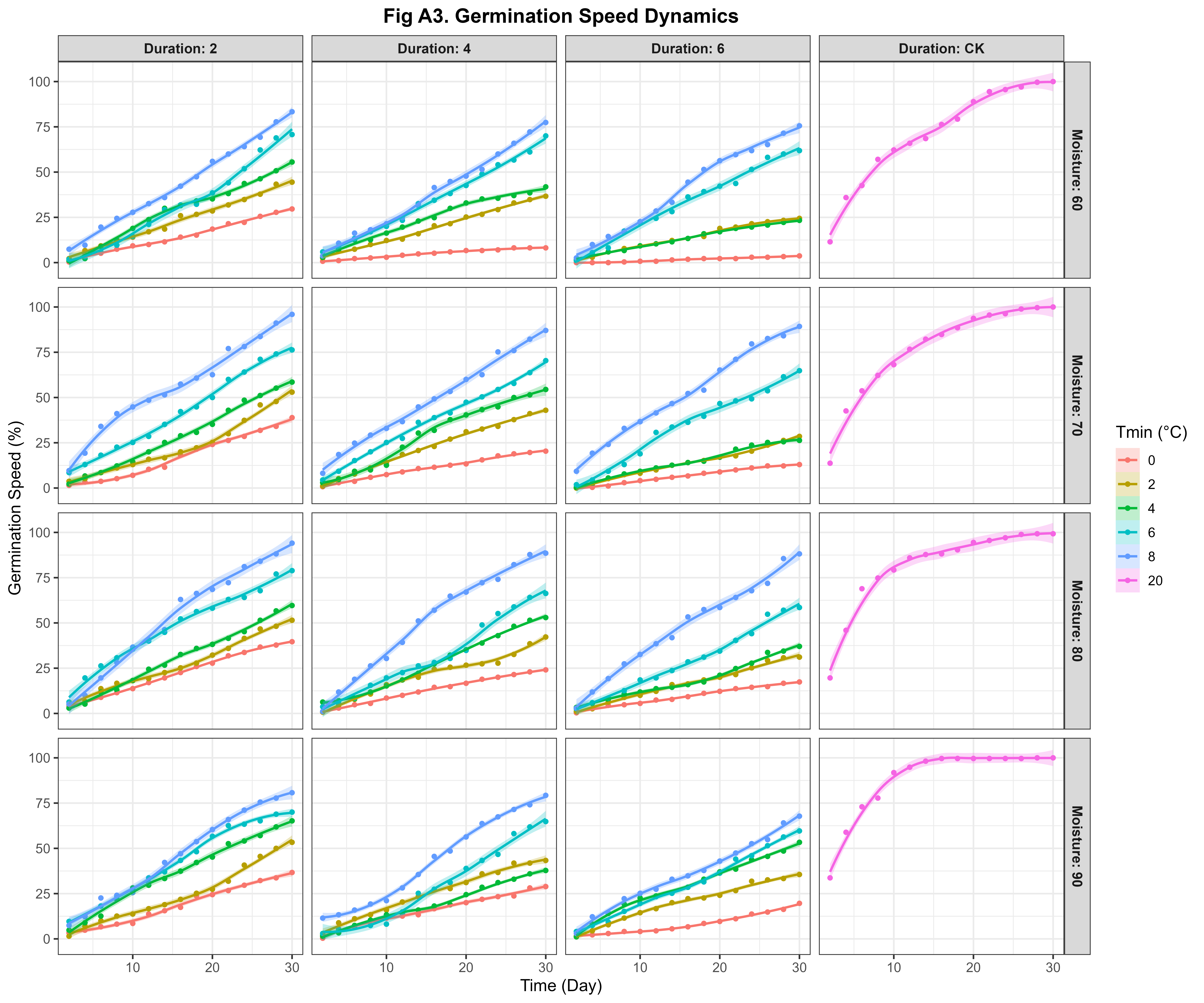

Supplement: S3 Fig A3 — Temporal trends of daily germination speed (%) under varying chilling durations (2,4,6 h and CK) and soil moisture levels. Loess curves illustrate average emergence rates, with shaded areas representing 95% confidence intervals. Color indicates Tmin level. (TIFF) [file pone.0340773.s003.tiff]

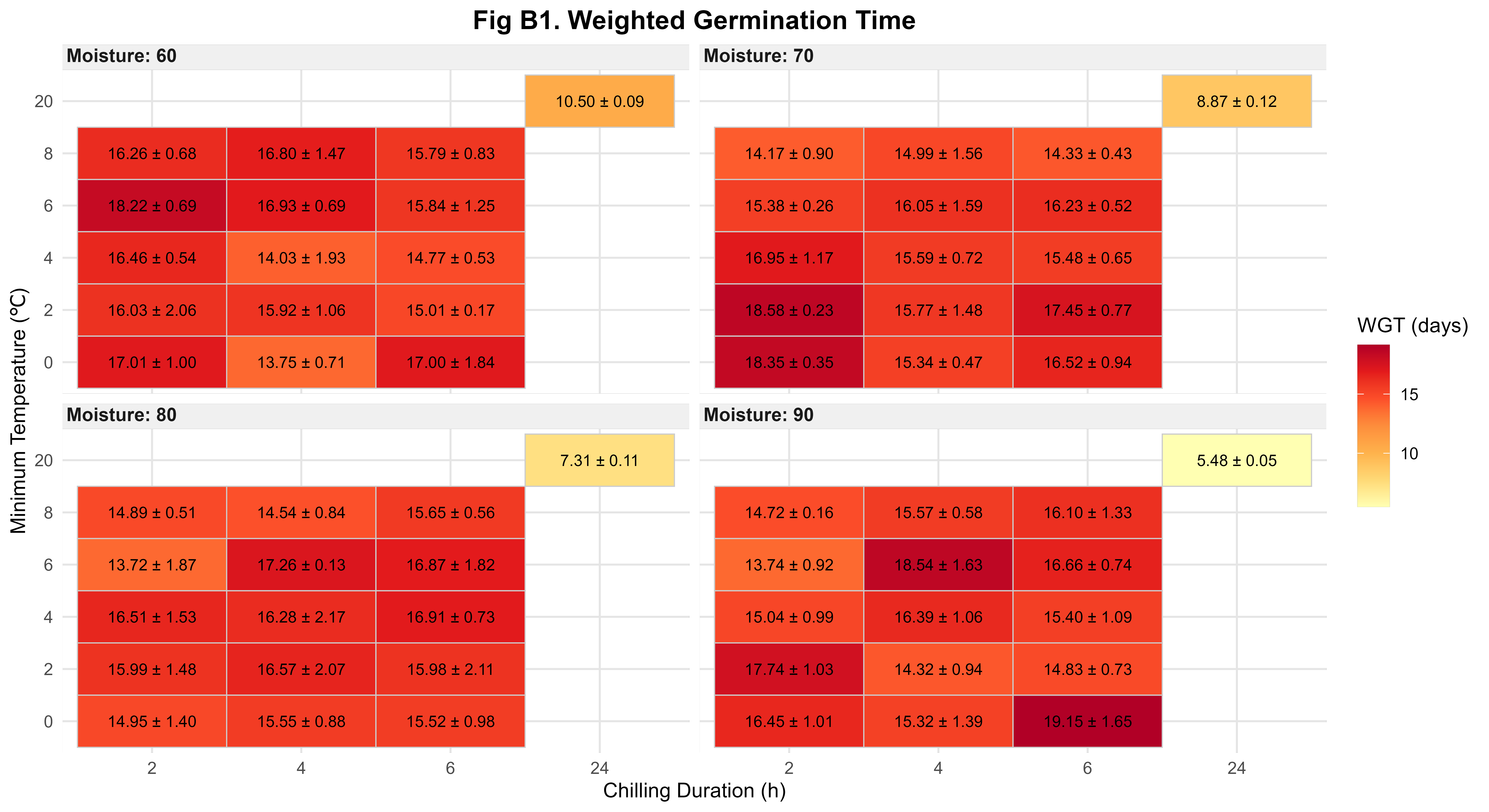

Supplement: S4 Fig B1 — WGT values across treatments of Tmin, chilling duration, and soil moisture levels. Higher Tmin and moisture resulted in earlier average emergence timing. Error bars denote standard error (SE) of mean values across replicates. (TIFF) [file pone.0340773.s004.tiff]

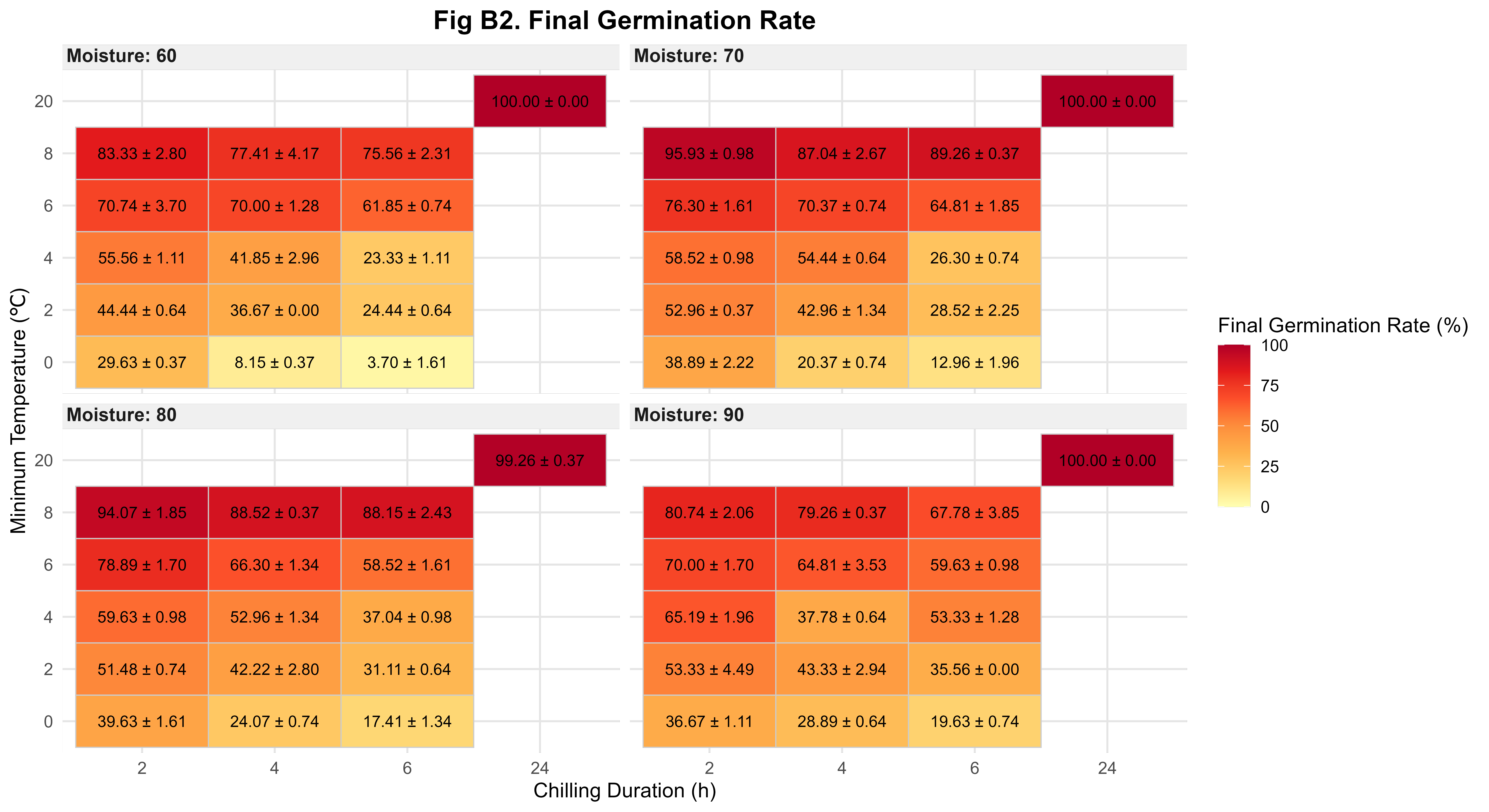

Supplement: S5 Fig B2 — Final germination rate (%) as influenced by Tmin, chilling duration, and soil moisture content. Data are mean±SE(n = 3). Higher Tmin and moisture supported better germination under chilling exposure. (TIFF) [file pone.0340773.s005.tiff]

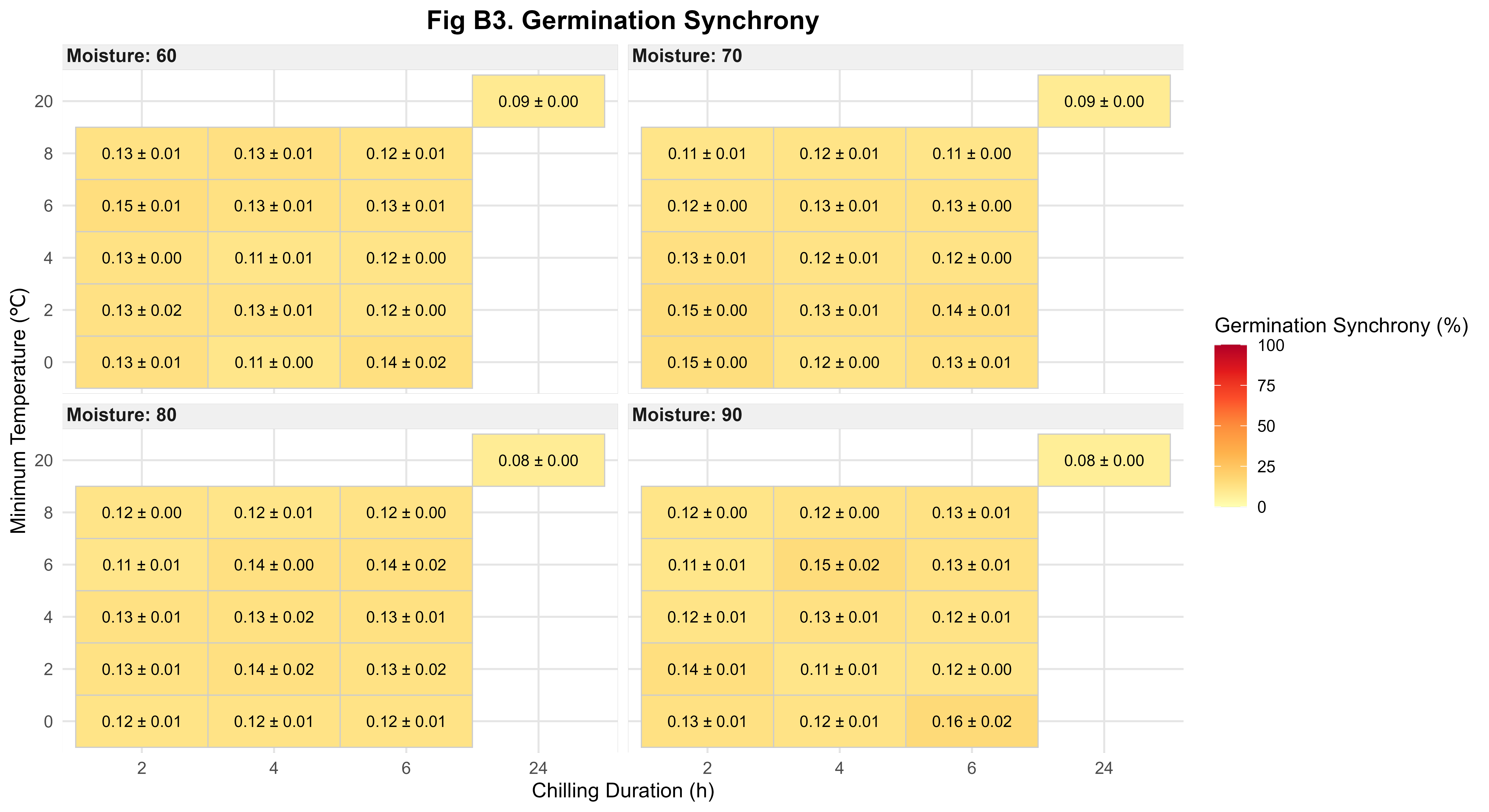

Supplement: S6 Fig B3 — Synchrony (%) of maize germination under different Tmin, chilling durations, and soil moisture levels.Synchrony is calculated as the peak emergence proportion relative to total emergence. Values represent mean ± SE. (TIFF) [file pone.0340773.s006.tiff]

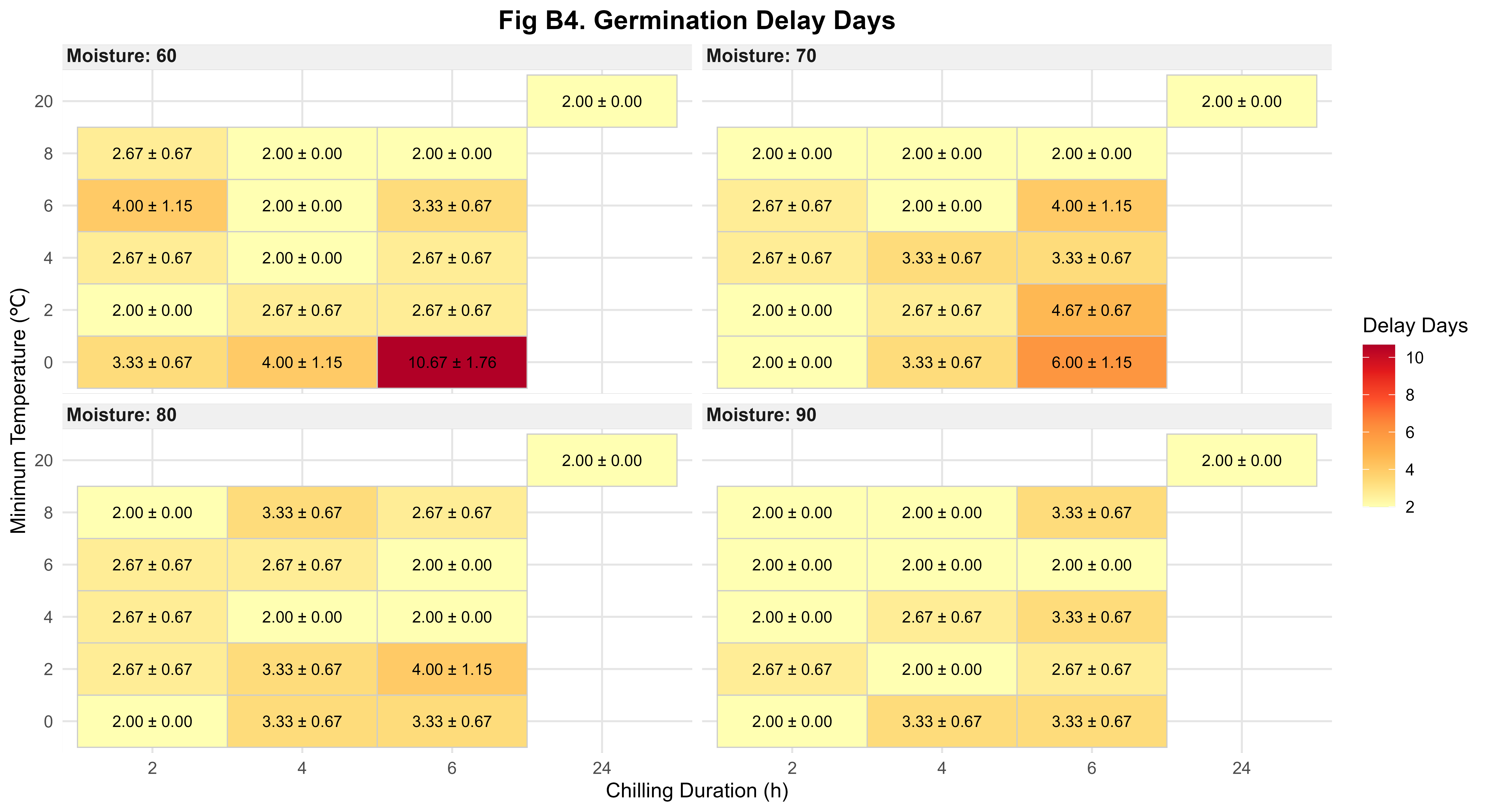

Supplement: S7 Fig B4 — Mean number of days until first emergence under varying Tmin,duration,and soil moisture levels.Greater delays occurred at Tmin≤2°C and low moisture.Bars represent mean±SE. (TIFF) [file pone.0340773.s007.tiff]

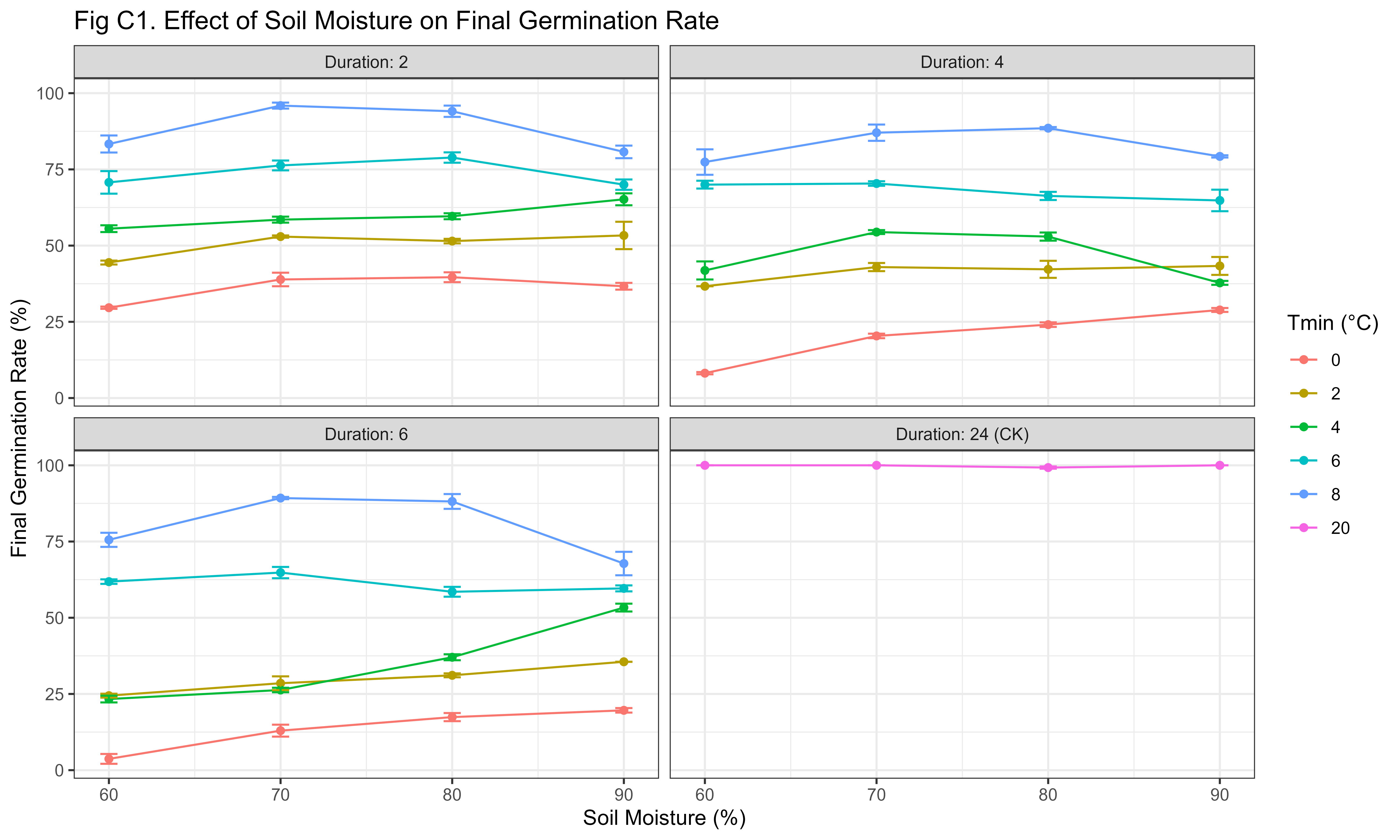

Supplement: S8 Fig C1 — Final germination rate (%) of maize seeds under soil moisture levels of 60–90% field capacity and chilling durations of 2, 4, 6, and 24 h. Increased soil moisture significantly enhanced final germination rate across all chilling durations. (TIFF) [file pone.0340773.s008.tiff]

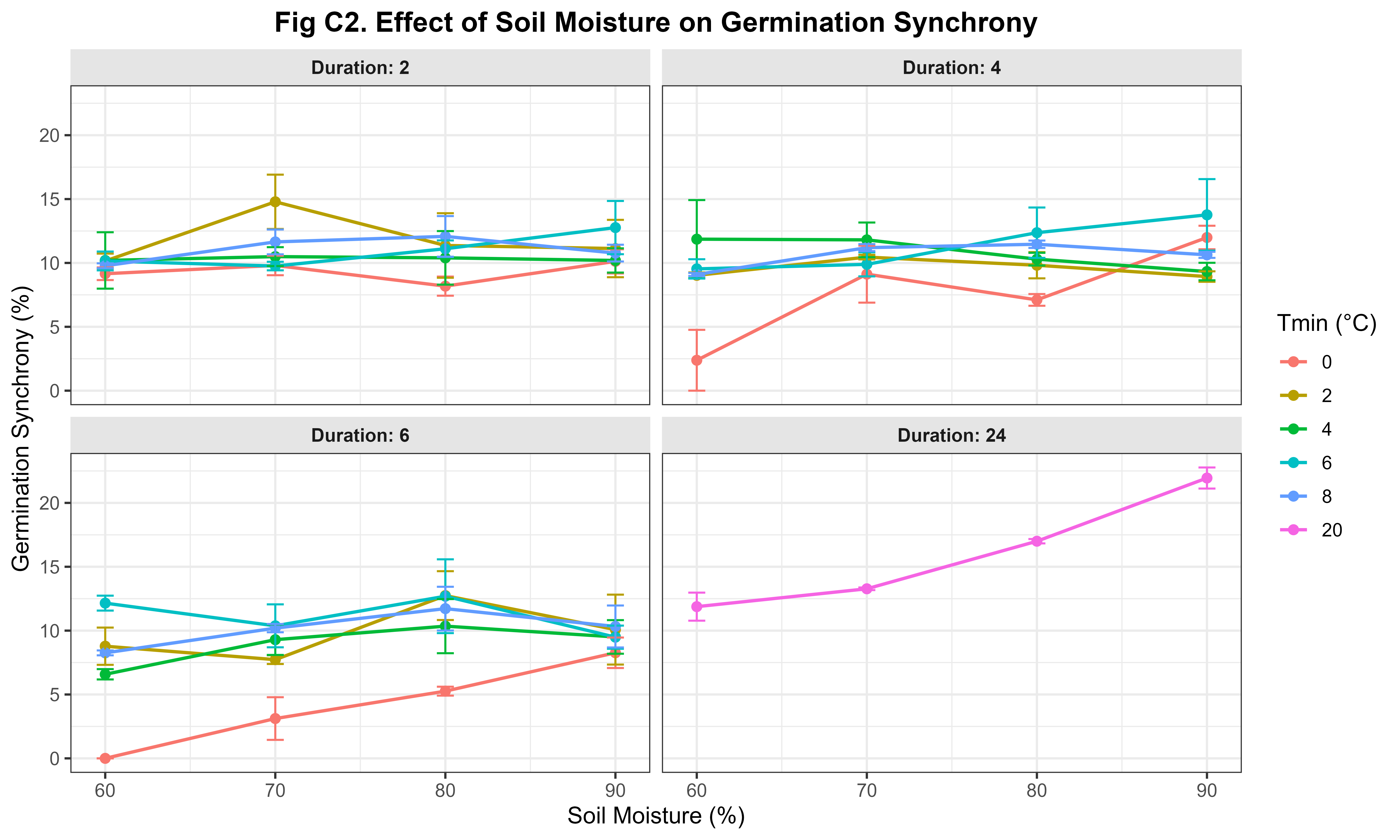

Supplement: S9 Fig C2 — Germination synchrony (%) of maize seeds at 60–90% field capacity subjected to chilling durations of 2, 4, 6, and 24 h. Higher soil moisture promoted more synchronized germination, particularly under prolonged chilling stress. (TIFF) [file pone.0340773.s009.tiff]

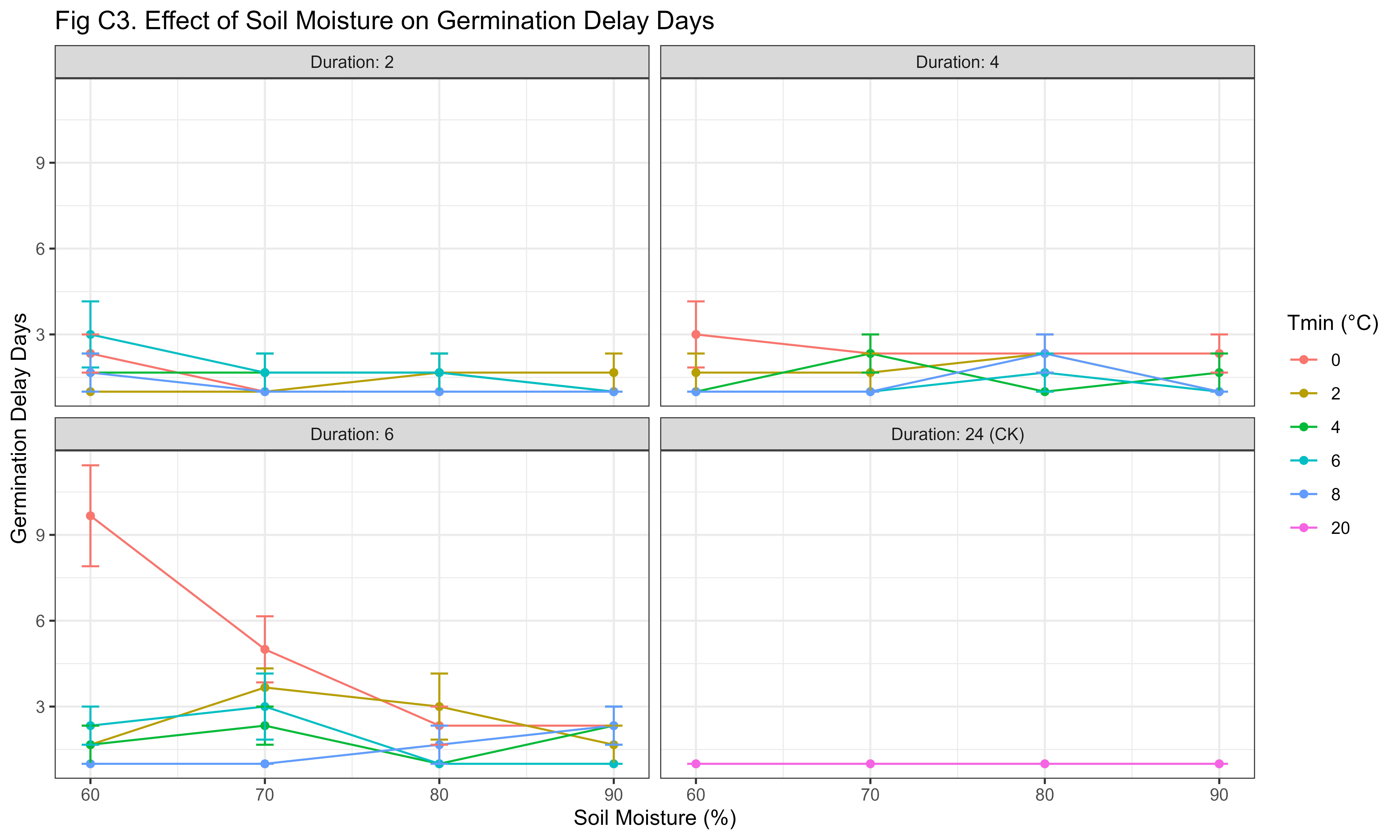

Supplement: S10 Fig C3 — Germination delay (days) of maize seeds under varying soil moisture conditions (60–90% field capacity) and chilling durations (2, 4, 6, and 24 h). Increasing soil moisture effectively reduced germination delay under all chilling treatments. (TIFF) [file pone.0340773.s010.tiff]

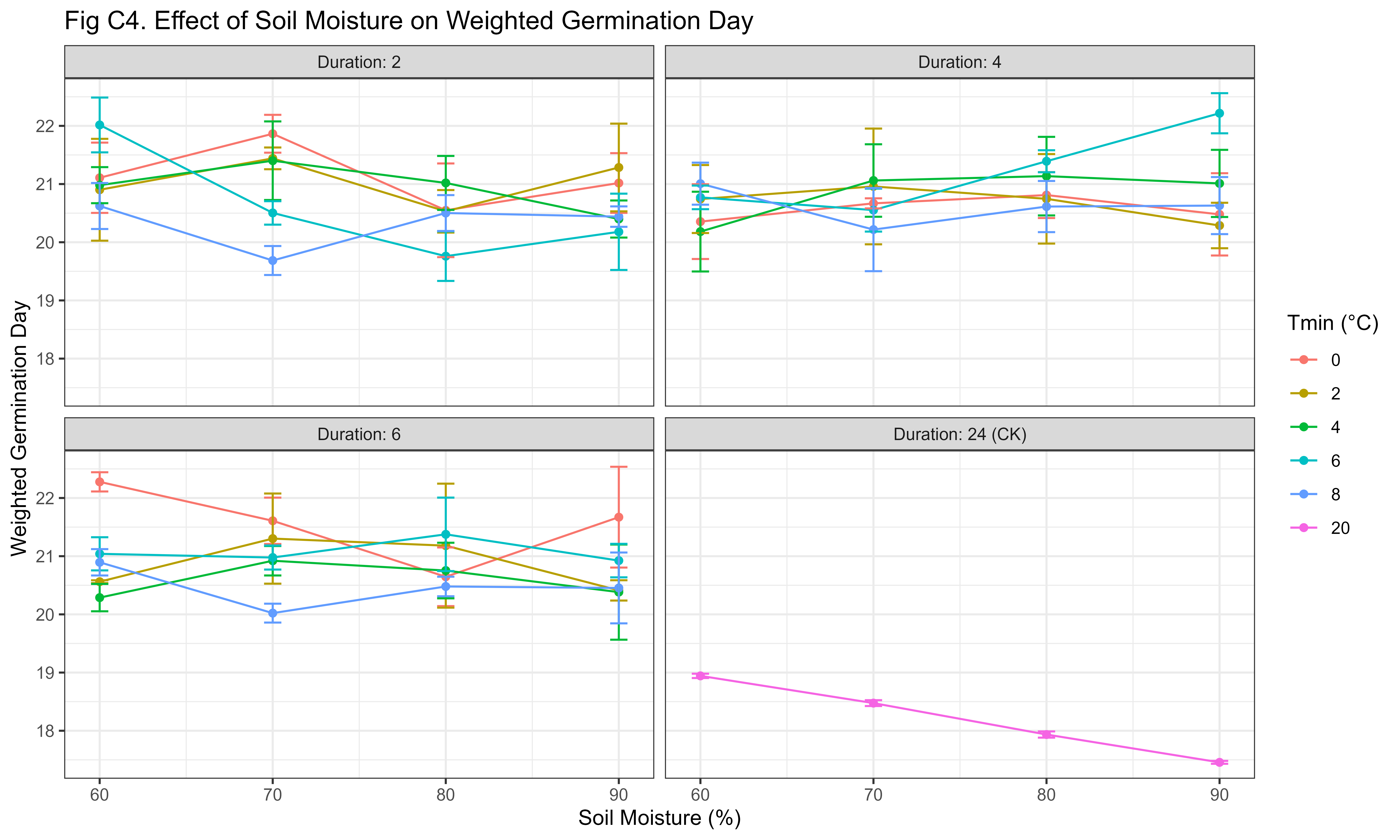

Supplement: S11 Fig C4 — Weighted germination time (days) of maize seeds under soil moisture levels of 60–90% field capacity and chilling durations of 2, 4, 6, and 24 h. Elevated soil moisture shortened weighted germination time, indicating improved germination performance under chilling stress. (TIFF) [file pone.0340773.s011.tiff]

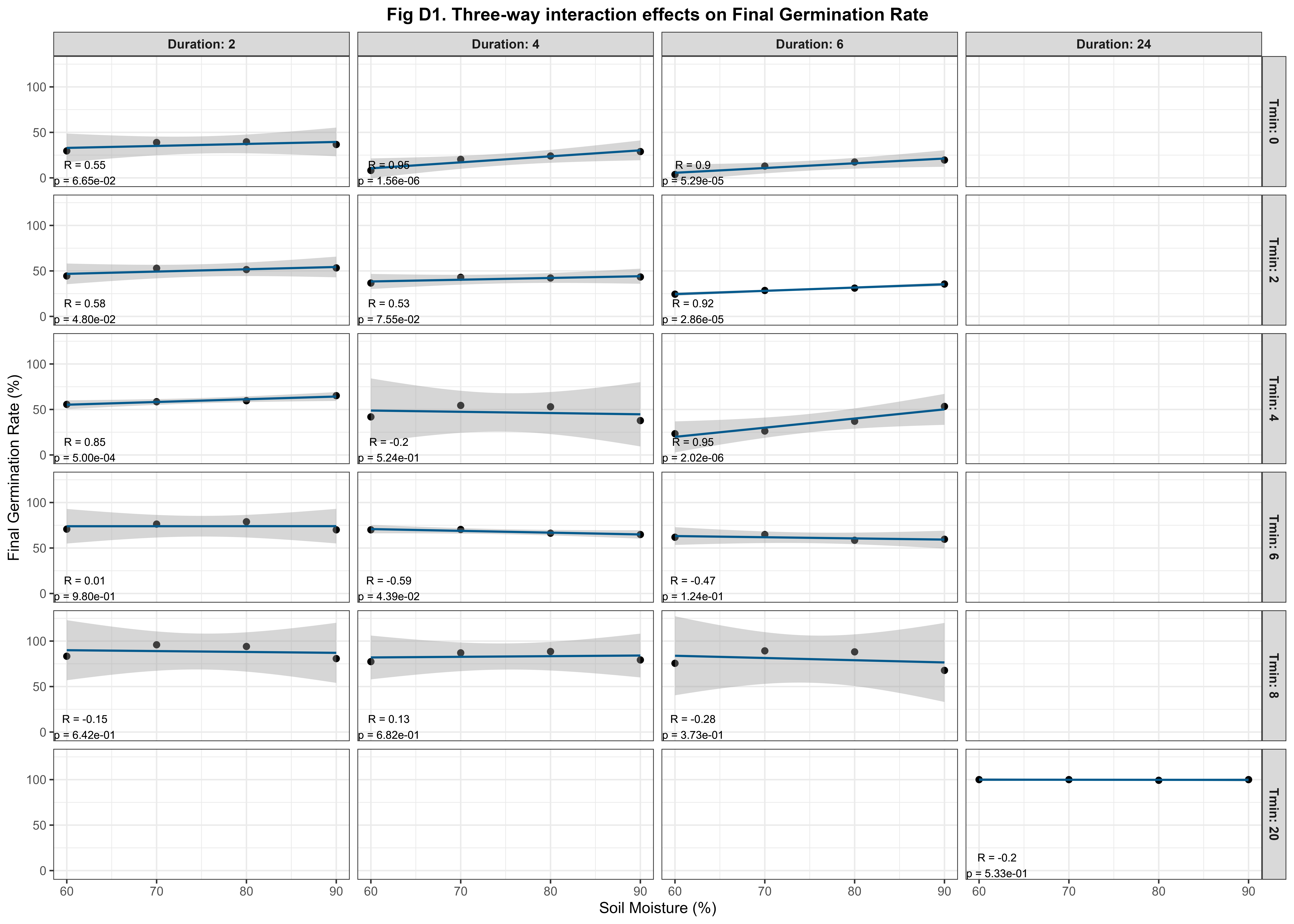

Supplement: S12 Fig D1 — Final germination rate (%) of maize seeds across combined treatments of minimum temperature (Tmin), chilling duration, and soil moisture. Lines represent means ± SE. Correlation coefficients (R) and corresponding p values are shown in each panel to quantify the relationships among the interactive factors. (TIFF) [file pone.0340773.s012.tiff]

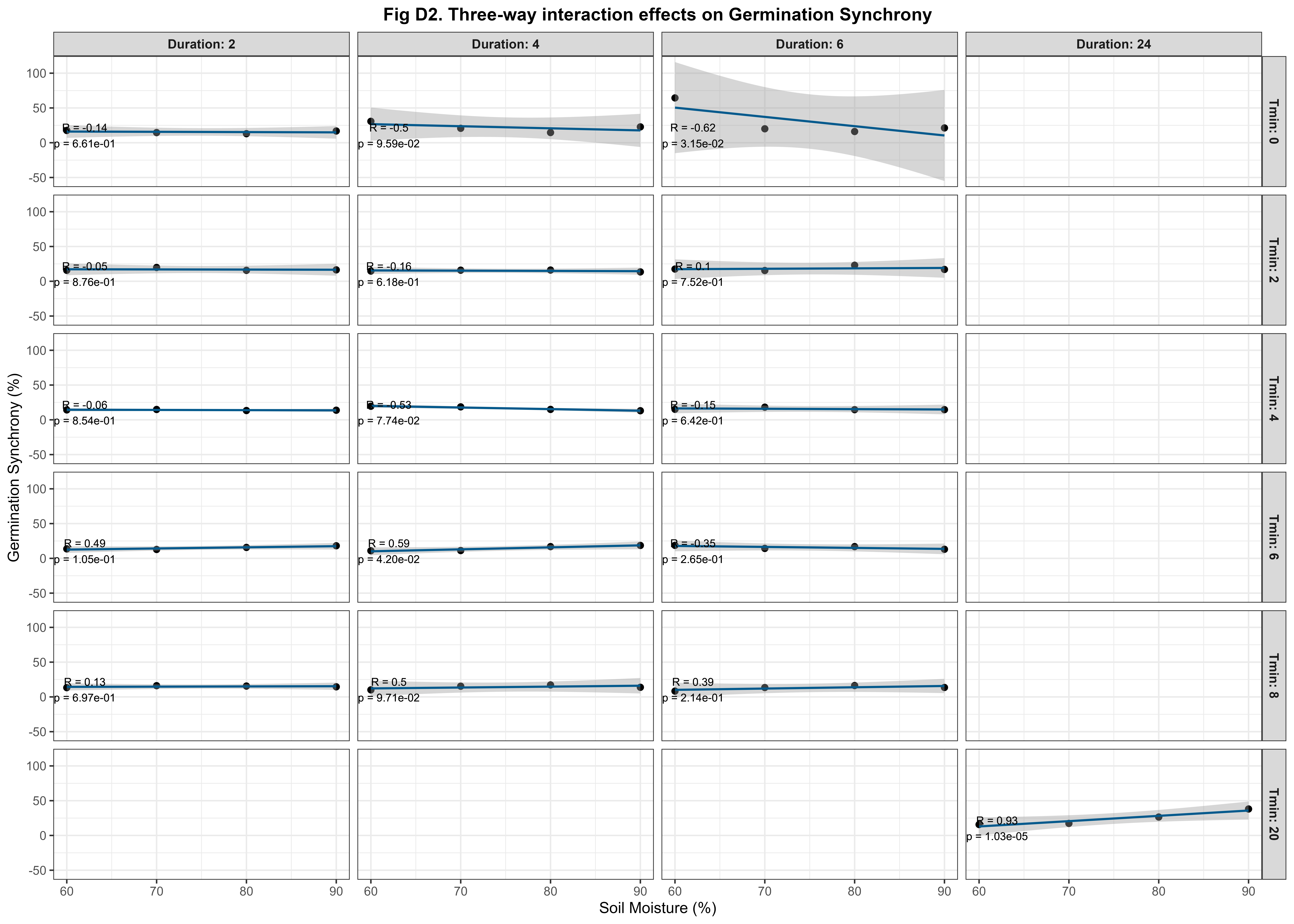

Supplement: S13 Fig D2 — Germination synchrony (%) of maize seeds under different combinations of Tmin, chilling duration, and soil moisture. Values are presented as means ± SE. The strength and significance of correlations are indicated by R and p values displayed within each panel. (TIFF) [file pone.0340773.s013.tiff]

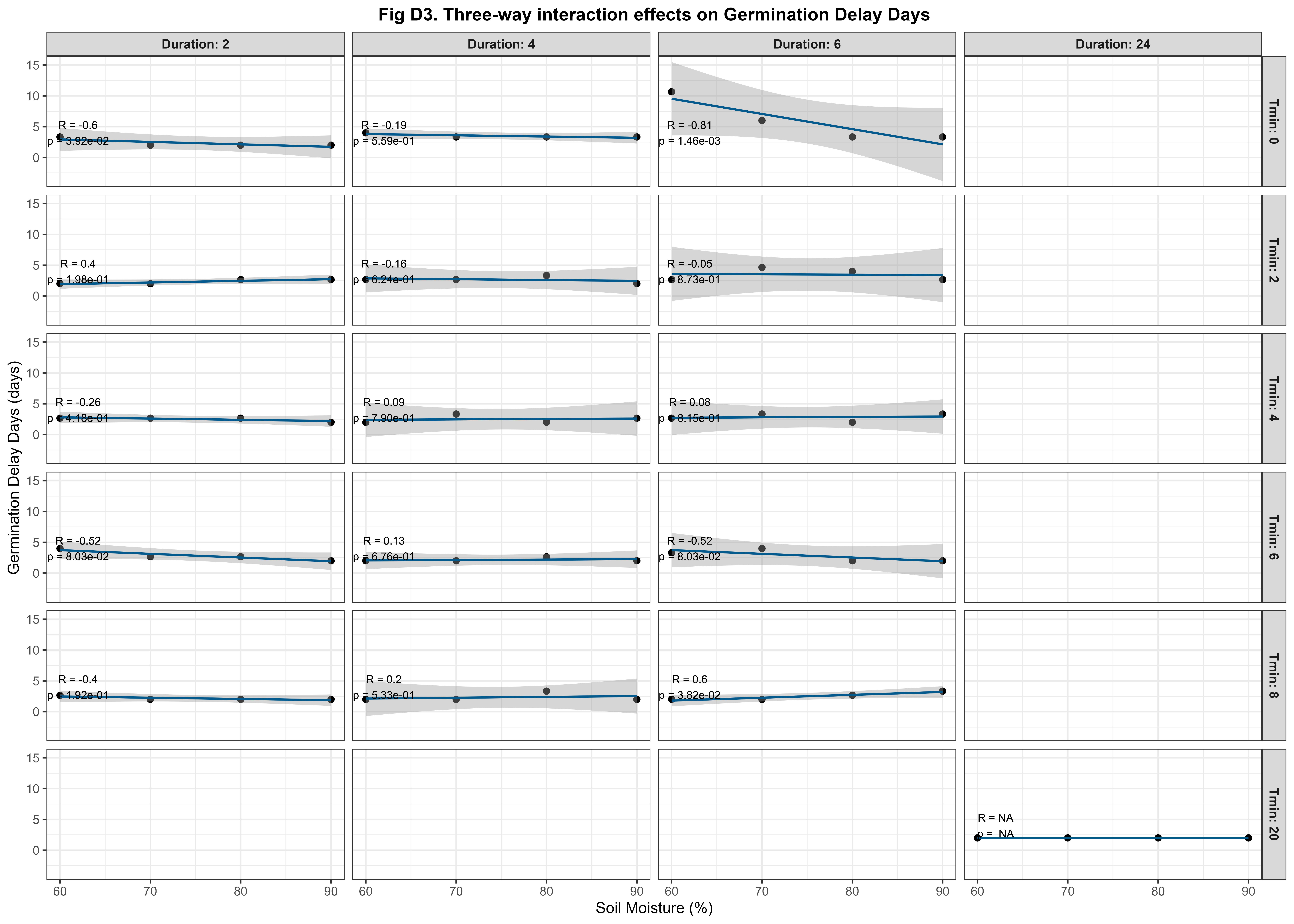

Supplement: S14 Fig D3 — Germination delay (days) of maize seeds in response to the interactive effects of Tmin, chilling duration, and soil moisture. Lines denote means ± SE, with correlation analysis results (R and p values) provided in each panel. (TIFF) [file pone.0340773.s014.tiff]

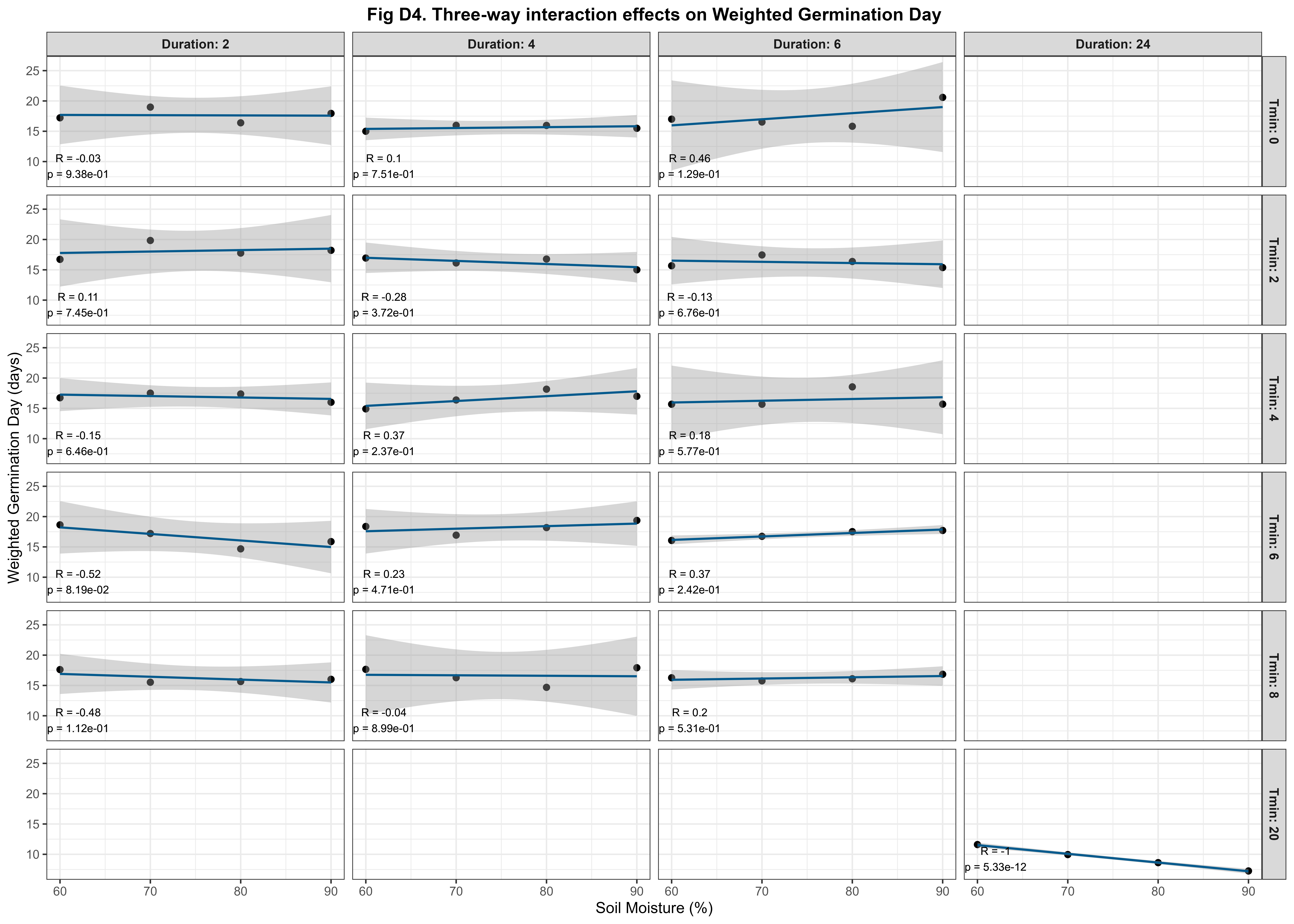

Supplement: S15 Fig D4 — Weighted germination time (days) of maize seeds across combinations of Tmin, chilling duration, and soil moisture. Data are shown as means ± SE, and correlation coefficients (R) and p values are reported within each panel. (TIFF) [file pone.0340773.s015.tiff]

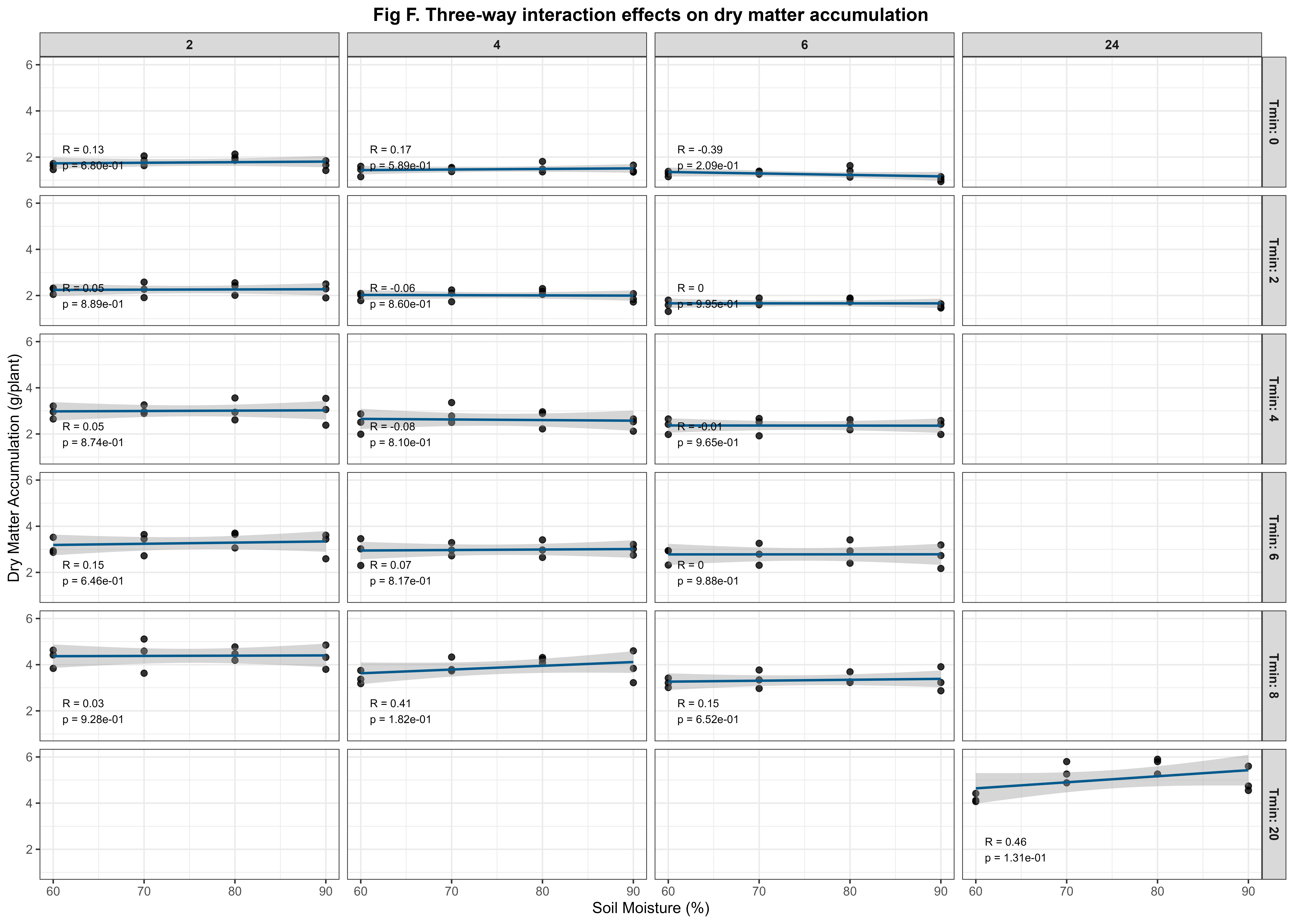

Supplement: S16 Fig F — Shoot dry weight (g plant ⁻ ¹) of maize seedlings at 30 days after sowing. Points represent individual replicates; regression lines indicate trends across moisture levels under different Tmin and chilling durations. ANOVA summary provided in S1 Table. (TIFF) [file pone.0340773.s016.tiff]
