# Supplementary material for: Moisture as a key factor alleviating low-temperature stress: Effects of hydrothermal conditions on maize emergence
Source: PLoS One. 2026 Feb 23;21(2):e0340773. doi: 10.1371/journal.pone.0340773 (PMC12928403; doi:10.1371/journal.pone.0340773)
Supplement: S1 Table — DFn = degrees of freedom numerator; DFd = degrees of freedom denominator;F = F-value; p = p-value; ges = generalized eta squared.Bolded p-values indicate statistical significance (p < 0.05). (PDF) [file pone.0340773.s017.pdf]

Table 1. Three-way ANOVA summary for the effects of Tmin, chilling duration, and soil moisture on maize shoot dry matter accumulation.

Effect	DFn	DFd	F	p	p<.05	ges
Tmin	4	128	243.86	7.69×10^{-59}	*	0.884
Duration	2	128	52.317	2.48×10^{-17}	*	0.45
Moisture	3	128	6.982	2.18×10^{-4}	*	0.141
Tmin:Duration	8	128	1.396	0.204		0.08
Tmin:Moisture	12	128	0.217	0.997		0.02
Duration:Moisture	6	128	0.15	0.989		0.007
Tmin:Duration:Moisture	24	128	0.257	1		0.046

Caption:DFn = degrees of freedom numerator; DFd = degrees of freedom denominator;
F = F-value; p = p-value; ges = generalized eta squared.
Bolded p-values indicate statistical significance ($p < 0.05$).
