# Supplementary material for: Moisture as a key factor alleviating low-temperature stress: Effects of hydrothermal conditions on maize emergence
Source: PLoS One. 2026 Feb 23;21(2):e0340773. doi: 10.1371/journal.pone.0340773 (PMC12928403; doi:10.1371/journal.pone.0340773)
Supplement: Attachment — Submitted filename: Peer_Review_Report_Maize_Chilling_Study.pdf [file pone.0340773.s018.pdf]

📝 Final Peer Review Report

Title of Manuscript: Interactive Effects of Chilling Temperature, Duration, and Soil Moisture on Maize Germination and Early Seedling Growth

Type of Article: Original Research

Recommendation: *Accept with minor revisions*

✅ Strengths of the Manuscript:

1. **Robust factorial design and statistical rigor:** The study employs a full factorial randomized block design with three key environmental factors (Tmin, chilling duration, and soil moisture), analyzed via three-way ANOVA and Pearson correlations. This provides strong statistical grounding.
2. **Timely and relevant topic:** The focus on early-season cold stress in semi-arid regions aligns well with current climate variability challenges and agronomic needs.
3. **Physiological depth and mechanistic insight:** The manuscript goes beyond descriptive results, offering mechanistic interpretations involving ROS balance, hormonal signaling, and membrane integrity under chilling stress.
4. **Use of advanced germination metrics:** Indices such as GI, GS, WGT, synchrony, and dry matter accumulation at day 30 provide a comprehensive view of germination dynamics and early vigor.
5. **Practical recommendations and modeling potential:** The study proposes moisture thresholds (≥80% FC) for mitigating chilling damage and suggests hydrothermal modeling as a tool for precision sowing strategies.

⚠ Points for Improvement and Clarification:

#	Topic	Comment & Suggestion
1	**Irrigation water quality**	The manuscript does not specify the type or quality of irrigation water used (e.g., EC, salt content). Clarifying this would improve reproducibility and relevance, especially in semi-arid regions prone to salinity.
2	**Diurnal temperature cycle logic**	The temperature cycle is mathematically constructed (T1 → Tmin → T1 → T2 → Tmax → T2), but it is unclear whether this pattern reflects actual field conditions. Authors should clarify whether it mimics natural spring temperature rhythms in Qiqihar.
3	**Field capacity levels**	The study uses 60–90% FC but omits 100% FC. Authors should briefly explain why full field capacity was excluded, as it may represent optimal water availability under non-stress conditions.
4	**Realistic field conditions**	In the Qiqihar region, during early spring maize sowing, snowmelt and initial seasonal rainfall may bring soil moisture levels close to saturation or even above field capacity. It is suggested that the authors briefly acknowledge this climatic reality so that readers can better contextualize the initial laboratory conditions of the study in

#	Topic	Comment & Suggestion
		relation to actual field scenarios. This is offered purely as a supplementary perspective and does not constitute a critique.
5	**Textual redundancy (Page 14)**	On page 14, two consecutive paragraphs describing the effects of chilling duration and soil moisture are highly similar. These could be merged or streamlined to improve clarity and avoid repetition.
6	**Suboptimal placement of temperature cycle description**	The detailed explanation of the six-phase temperature cycle appears in the "Experimental Conditions" section. This technical content would be better placed under a separate subheading (e.g., “Temperature Simulation Protocol”) for improved structural flow.
7	**Substrate type**	The use of sterilized sand as a growth medium may limit extrapolation to field soils. A brief note on this limitation would strengthen the discussion.
8	**Post-germination growth stages**	The study ends at day 30 with dry matter measurement. While this is sufficient for current scope, authors may suggest future work on later vegetative stages (e.g., V3, V5) to assess long-term recovery. This is a recommendation for future research, not a critique.
9	**Farmer behavior and sowing timing**	In the Qiqihar region, maize sowing typically begins in early spring, even under conditions of low temperature or high soil moisture. It may be helpful for the authors to briefly discuss what limiting or compelling factors prevent farmers from delaying sowing by a few days to reduce germination risks. These could include agronomic calendar constraints, labor availability, or economic pressures. Such context would help readers better understand how the experimental findings relate to real-world decision-making in the field. This is offered purely as a supplementary perspective and does not constitute a critique.
10	**Actionable advice for farmers**	Authors are encouraged to include a practical summary or table of recommended Tmin–moisture combinations to guide sowing decisions under variable spring conditions.

🏁 Final Recommendation:

The manuscript is scientifically sound, timely, and well-executed. With minor revisions addressing the points above, it will be suitable for publication in a high-quality agronomy or plant physiology journal.
